# New mechanistic insights into the RAS-SIN1 interaction at the membrane

**DOI:** 10.3389/fcell.2022.987754

**Published:** 2022-10-06

**Authors:** Silke Pudewell, Jana Lissy, Hossein Nakhaeizadeh, Niloufar Mosaddeghzadeh, Saeideh Nakhaei-Rad, Radovan Dvorsky, Mohammad R. Ahmadian

**Affiliations:** ^1^ Institute of Biochemistry and Molecular Biology II, Medical Faculty and University Hospital Düsseldorf, Heinrich Heine University Düsseldorf, Düsseldorf, Germany; ^2^ Stem Cell Biology and Regenerative Medicine Research Group, Institute of Biotechnology, Ferdowsi University of Mashhad, Mashhad, Iran; ^3^ Center for Interdisciplinary Biosciences, P. J. Šafárik University, Košice, Slovakia

**Keywords:** RAS, RAS family, SIN1, MAPKAP1, mTORC2, ras binding domain, PH domain, membrane binding

## Abstract

Stress-activated MAP kinase-interacting protein 1 (SIN1) is a central member of the mTORC2 complex that contains an N-terminal domain (NTD), a conserved region in the middle (CRIM), a RAS-binding domain (RBD), and a pleckstrin homology domain. Recent studies provided valuable structural and functional insights into the interactions of SIN1 and the RAS-binding domain of RAS proteins. However, the mechanism for a reciprocal interaction of the RBD-PH tandem with RAS proteins and the membrane as an upstream event to spatiotemporal mTORC2 regulation is not clear. The biochemical assays in this study led to the following results: 1) all classical RAS paralogs, including HRAS, KRAS4A, KRAS4B, and NRAS, can bind to SIN1-RBD in biophysical and SIN1 full length (FL) in cell biology experiments; 2) the SIN1-PH domain modulates interactions with various types of membrane phosphoinositides and constantly maintains a pool of SIN1 at the membrane; and 3) a KRAS4A-dependent decrease in membrane binding of the SIN1-RBD-PH tandem was observed, suggesting for the first time a mechanistic influence of KRAS4A on SIN1 membrane association. Our study strengthens the current mechanistic understanding of SIN1-RAS interaction and suggests membrane interaction as a key event in the control of mTORC2-dependent and mTORC2-independent SIN1 function.

## 1 Introduction

Mammalian target of rapamycin complexes (mTORC) one and two are key regulators of many cellular processes in response to a broad spectrum of extracellular stimuli (Brown et al., 1994; Huang and Fingar, 2014; Saxton and Sabatini, 2017). mTORC1 mediates the control of cell growth through the activation of anabolic processes, whereas mTORC2 facilitates the spatial control of cell survival, cell growth, and actin cytoskeleton organization through the phosphorylation of AGC family protein kinases, including AKT, SGK, and PKC (Loewith et al., 2002; Jacinto et al., 2004; Pearce, Komander and Alessi, 2010; Saxton and Sabatini, 2017). The catalytic subunit of both complexes is mTOR which contains a serine/threonine kinase domain.

The stress-activated MAP kinase-interacting protein 1 (SIN1) is one of the four conserved components of the mTORC2 complex, which consists of SIN1, mTOR, mLST8, RICTOR and can associate with the accessory proteins PROTOR and DEPTOR (Oh and Jacinto, 2011). Little is known about the upstream regulators of mTORC2 but it is shown that its regulation and activity depend on its subcellular localization and it is found in multiple pools in the cytosol, plasma membrane, early and late endosomes, and mitochondria (Ebner et al., 2017; Fu and Hall, 2020). The activity of the mTORC2 complex specifically depends on its components (Chen et al., 2018; Stuttfeld et al., 2018; Scaiola et al., 2020; Tafur, Kefauver and Loewith, 2020). MLST8 functions as a scaffold to maintain mTORC2 integrity and kinase activity (Hwang et al., 2019), whereas RICTOR acts as an essential core for mTORC2 complex formation (Gao et al., 2021). The role of PROTOR as a novel RICTOR-binding subunit of mTORC2 is yet unclear (Pearce et al., 2007). DEPTOR appears to block mTORC2 activity (Peterson et al., 2009), a process that is prevented by its tyrosine phosphorylation (Gagné et al., 2021). SIN1 is required for mTORC2 activity and may function by regulating mTOR association with membranes (Frias et al., 2006; Yang et al., 2006; Liu et al., 2013, 2014; Yuan et al., 2015; Yuan and Guan, 2015). SIN1-NTD interacts tightly with RICTOR and mLST8 in an extended conformation and links RICTOR to mLST8 (Scaiola et al., 2020). The increase in RICTOR ubiquitination prevented RICTOR and mSIN1 from interacting with mTOR while leaving the interaction between RICTOR and mSIN1 unaffected (Wrobel et al., 2020). In contrast to NTD and CRIM domains, RBD and PH domains of SIN1 remain flexibly disposed of this complex (Scaiola et al., 2020).

Phosphoinositide 3-kinase (PI3K)-dependent activation is partially executed at the plasma membrane in response to extracellular growth factors, which can trigger the recruitment of the effector AKT to the membrane. Insulin-PI3K signaling induces furthermore the association of mTORC2 with ribosomes, which activates the complex and may be part of the co-translational phosphorylation of AKT and PKC (Oh et al., 2010; Zinzalla et al., 2011). The role of SIN1 in the regulation and activation of mTORC2 is complex and predominantly involves the RAS-binding domain (RBD) and the pleckstrin homology (PH) domain. The other two domains, the N-terminal domain (NTD) and the conserved region in the middle (CRIM) domain, are responsible for interactions with RICTOR and mTORC2 substrate recognition, respectively (Tatebe et al., 2017). The PH domain of SIN1 binds to phosphatidylinositol (3,4,5)-triphosphate (PIP_3_) and therefore relives the inhibitory binding of the PH domain on mTOR that initially masks the catalytic pocket of the complex (Liu et al., 2015; Yuan and Guan, 2015). The RBD of SIN1 raised many questions during the past years. SIN1 binding to HRAS and KRAS reduced RAS signaling toward ERK after its overexpression, while higher ERK activity was observed under SIN1 knockdown conditions (Schroder et al., 2007). Castel *et al.* characterized the SIN1-RBD/RAS interaction and demonstrated a critical interaction of the guanine nucleotide-binding (G) domain and the C-terminal hypervariable region (HVR) of KRAS4A with a region of SIN1 (amino acids: 364-390), which was called an alternative RBD or aRBD (Castel et al., 2021). However, deletion of the aRBD had no impact on cell signaling or animal development based on their observations. Zheng *et al.* recently provided further structural insights into the SIN1-RBD interaction with HRAS (Zheng et al., 2022). They remarkably proposed an insulin-induced reduction of ERK phosphorylation as a result of the RAS-SIN1 interaction (Zheng et al., 2022).

To gain more insights into the SIN1-RBD function, additional analyses are required to understand the inter-domain relationship of the SIN1-RBD-PH tandem in the interaction with RAS proteins, the membrane, and its mechanistic role in the regulation of mTORC2 in response to growth factor stimulation. Therefore, we have examined the direct binding of SIN1-RBD with various small GTPases and the effect of the PH domain on RAS and membrane binding. Furthermore, we monitored the impact of different SIN1 constructs on the mTORC2-AKT and MAPK pathways.

## 2 Materials and methods

### 2.1 Reagents

Dulbecco’s modified Eagle’s medium (DMEM), fetal bovine serum (FBS), and penicillin/streptomycin were obtained from Gibco^®^ Life Technologies. The following antibodies were used: anti-α-tubulin (#ab52866, Abcam), anti-tAKT (#2920, Cell Signaling), anti-pAKT^S473^ (#4060, Cell Signaling), anti-pERK^T202/Y204^ (#9106, Cell Signaling), anti-FLAG (#F3165, Sigma Aldrich), anti-GAPDH (#398600, Invitrogen), anti-GST (own antibody), anti-γ-tubulin (#T5326, Sigma Aldrich), anti-His (#MA5-33032, Thermo Fisher), anti-KRAS (#12063-1-AP, Proteintech), anti-NRAS (#EB08365, Erest Biotech) and anti-SIN1 (#2746272, Merck Millipore). The secondary antibodies IRDye^®^ 800CW donkey anti-rabbit IgG and IRDye^®^ 680RD donkey anti-mouse IgG were purchased from Li-Cor and analyzed in the Odyssey^®^ XF Imaging System. The nucleotides mGDP (methylanthraniloyl- or mant-GDP), mGppNHp (mant-GppNHp) and GppNHp were obtained from Jena Bioscience GmbH. Human EGF and GDC-0941 were obtained from Merck (Darmstadt, Germany).

### 2.2 Constructs and proteins

Full length genes of RAS and RHO GTPases (Table 1) were cloned into pGEX-4T1-N-Tev vectors and purified from *Escherichia coli* using glutathione-based affinity chromatography and size exclusion chromatography as described previously (Gremer et al., 2011). Full length *SIN1* (Q9BPZ7), *SIN1* domains (RBD: aa 266-373; RBD-PH: aa 266-522; PH: aa 373-522), *PI3Kα-RBD, RAF1-RBD* and *SIN1* mutants (RBD^K307D^, RBD^RR311-312EE^ and RBD^FSL289-291REE^) were cloned and expressed in pGEX-4T1-N-Tev or pMal-c5X-His and purified by glutathione- or Ni-NTA-based affinity chromatography and size exclusion chromatography, as previously described (Hemsath and Ahmadian, 2005; Gremer et al., 2011). *KRAS4A* was further cloned into the pFAST-Bac vector for expression and purification from insect cells as described previously (Zhang et al., 2014). *SIN1 FL*, isoform 6 (Q9BPZ7-6), ΔaRBD (aa 363-390 missing), and the RAS GTPases *HRAS* (P01112), *NRAS* (P01111), *KRAS4A* (P01116-1), *KRAS4B* (P01116-2), *RIT1* (Q92963) and *ERAS* (Q7Z444) were cloned into pcDNA-3.1-FLAG, pcDNA-3.1 (-) or pEYFP for eukaryotic expression. These vectors were provided by Alfred Wittinghofer of the Max Planck Institute Dortmund.

**TABLE 1:** SIN1-RBD interaction with proteins of the RAS superfamily.

Protein	K_d_ (µM)	Uniprot ID
HRAS	24 ± 2	P01112
NRAS	31 ± 2	P01111
KRAS4B	33 ± 2	P01116-2, P011118-1
KRAS4A	34 ± 1	P01116-1
RIT1	123 ± 15	Q92963
RRAS	123 ± 18	P10301
ERAS	171 ± 15	Q7Z444
RALA	331 ± 61	P11233
RHEB	358 ± 48	Q15382
RAP2A	483 ± 91	P10114
RHOA	535 ± 231	P61586
TC21	878 ± 472	P62070
CDC42	No binding observed	P60953
RAC1	No binding observed	P63000
RAC2	No binding observed	P15153

Values displayed are K_d_ ± SD, in µM.

### 2.3 Cell culture, transfection and cell lysis

HEK293 and COS7 cells were cultured in DMEM supplemented with 10% FBS and 1% penicillin/streptomycin. Transfection was performed using TurboFect™ Transfection Reagent (Thermo Fisher Scientific) following the manufacturer’s protocol. Cells were lysed in FISH buffer containing 50 mM Tris/HCl (pH 7.5), 100 mM NaCl, 2 mM MgCl_2_, 10% glycerol, 20 mM *ß*-glyerolphosphate, 1 mM Na_3_VO_4_, 1x protease inhibitor cocktail and 1% IGPAL on ice for 10 min and centrifuged for 10 min at 16,000 rpm.

### 2.4 CRISPR/Cas9 knock-out

CRISPR/Cas9 knockout was performed by incubating purified TrueCutTM Cas9 protein v2 (Thermo Fisher Scientific) with TrueGuideTM Synthetic sgRNA for human MAPKAP1 (Thermo Fisher Scientific, Assay ID: CRISPR1072864_SGM) in nucleofection solution SF (LONZA) for 30 min at room temperature. Then, 1*10^6^ HEK293 cells were resuspended in the solution and nucleofected in the 4D Nucleofector X-Unit (LONZA) using pulse CM-130. Cells were expanded for 1 week and then separated on 96-well plates to obtain single clones.

### 2.5 GST pull-down assay

Pull-down experiments were performed using purified GST-fused proteins coupled to glutathione agarose beads (Sigma Aldrich, Germany). Proteins were coupled for 1 h at 4°C on a rotor and centrifuged at 500 x g. The beads were washed 3 times with a cold buffer containing 50 mM Tris-HCl, 150 mM NaCl and 10 mM MgCl_2_ and incubated with COS7 or HEK293 cell lysates with endogenous or overexpressed proteins for 1 hour. The beads were washed 3 times, and the proteins were mixed with 1x Laemmli buffer. Samples were analyzed using SDS-PAGE and immunoblotting.

### 2.6 Immunoprecipitation

EYFP-HRAS^G12V^ was overexpressed in COS7 cells. Cell lysates were incubated overnight at 4°C with GFP nanobodies (GFP-binding domain of Lama single-chain antibody) covalently bound to Sepharose beads. The nanobody beads were washed three times, and the remaining protein was mixed with 1x Laemmli and analyzed by SDS-PAGE and immunoblotting as described previously (Rothbauer et al., 2008; Nakhaei-Rad et al., 2016). The nanobody beads were washed three times, and the remaining protein was mixed with 1x Laemmli and analyzed using SDS-PAGE and immunoblotting as described before.

### 2.7 Structural modeling of SIN1-RBD and its complex with HRAS

A structural model of the RBD from SIN1 was created with the computer program Modeler (https://www.ncbi.nlm.nih.gov/pmc/articles/PMC5031415/) using the automodel command. As a template, isolated SIN1-RBD and in complex with HRAS, the structure of RAF kinase RBD in complex with HRAS•GppNHp (PDB: 4G0N) was used. Final structures were refined *via* a short minimization of complex energy with the program CHARMm (https://www.ncbi.nlm.nih.gov/pmc/articles/PMC2810661/) using default parameters.

### 2.8 Fluorescence polarization

Fluorescence polarization experiments were executed *via* the titration of increasing amounts of the effector (SIN1) proteins to 1 µM mGppNHp- and mGDP-bound GTPases as described before (Gremer et al., 2011; Nakhaeizadeh et al., 2016). Experiments were performed using a Fluoromax 4 fluorimeter in polarization mode vs. time (excitation wavelength: 360 nm, emission wavelength: 450 nm), at 21°C in a buffer, containing 30 mM Tris/HCl pH 7.5, 150 mM NaCl, 5 mM MgCl_2_ and 3 mM dithiothreitol. The dissociation constants (K_d_) were calculated using a quadratic ligand binding equation in GraFit 5.

### 2.9 Liposome assays

Liposomes were prepared by mixing 10% (w/w) phosphatidylethanolamine (PE) (for flotation assay: 5% (w/w) PE and 5% (w/w) fluorescently labeled NBD-PE), 50% (w/w) phosphatidylcholine (PC), 20% (w/w) phosphatidylserine (PS), 5% (w/w) phosphatidylserine, 5% (w/w./wt.) phosphatidylinositol-3-monophosphate (PIP), 5% (w/w) phosphatidylinositol-4,5-bisphosphate (PIP2), and 5% phosphatidylinositol-3,4,5-triphosphate (PIP3) from stock solutions dissolved in chloroform. Negative liposomes (Supplementary Figure S7) were prepared by mixing 90% (w/w) and 10% (w/w) PE. The final mixture (500 µg) was dried and rehydrated in 500 µl buffer containing 20 mM HEPES (pH 7.5), 50 mM NaCl, 5 mM MgCl2, and 3 mM DTT. The solution was sonicated 10 times under mild conditions (minimum power, 50% on and 50% off) and extruded 21 times through a membrane with a pore size of 0.2 µm.

PIP strips were purchased from Echelon Bioscience and treated according to the manufacturer’s protocol. Briefly, the lipid-containing membrane was blocked for 1 hour with TBS containing 3% BSA (PanReac AppliChem GmbH). The SIN1-PH domain was incubated with the membrane at a concentration of 1 μg/ml in TBS +3% BSA for 1 hour. Three washing steps with TBS +0.1% Tween 20 were followed by a 1-h incubation with the appropriate primary antibody. The washing steps were repeated, and the membrane was incubated with the secondary antibody from Li-Cor for 1 hour. After three more washing steps, the membrane was evaluated in a Li-Cor Odyssey system.

Liposome sedimentation assays were performed by mixing 60 µl liposomes with 20 µl SIN1-PH (1-3 µM), incubating the sample for 30 min at 4°C while mixing followed by 30 min centrifugation at 20.000 *g* at 4°C. The supernatant and pellet were mixed or resuspended with 5x Laemmli to obtain a final volume of 92 µl. The samples were loaded on an SDS gel and analyzed using Coomassie staining or immunoblotting.

For the liposome flotation assay, 50 µl of liposomes (or negative liposomes; Supplementary Figure S8) (containing 5% fluorescently labeled NBD-PE) were mixed with 50 µl of SIN1 proteins (25 µM) and incubated at 4°C for 30 min. The sample was mixed with 100 µl of 60% sucrose and pipetted into a small centrifuge tube (Beckman Coulter). On top of the first layer, 250 µl of 25% sucrose and 50 µl of PBS −/− were added without allowing the phases to mix. The samples were centrifuged for 1 hour at 50,000 rpm at 4°C. The fluorophore-containing liposome phase was detected using a UV lamp and taken in a 50 µl total volume. Samples were evaluated as described for the liposome sedimentation assay.

### 2.10 Partial fractionation

Partial fractionation was performed using the Mem-PER™ Plus Membrane Protein-Extraction Kit (Thermo Fisher Scientific) following the manufacturer’s protocol. Briefly, cells were trypsinized and washed twice with a cell wash solution. The membrane was permeabilized with 375 µl permeabilization buffer for 10 min at 4°C and centrifuged for 15 min at 16,000 rpm. The cytosolic fraction was mixed with 5x Laemmli. The membrane pellet was resuspended in solubilization buffer and incubated for 30 min at 4°C while mixing. The sample was centrifuged for 15 min at 16,000 rpm, and the supernatant was mixed with 5x Laemmli. Samples were analyzed using SDS-PAGE and immunoblotting.

## 3 Results

**FIGURE 1:**
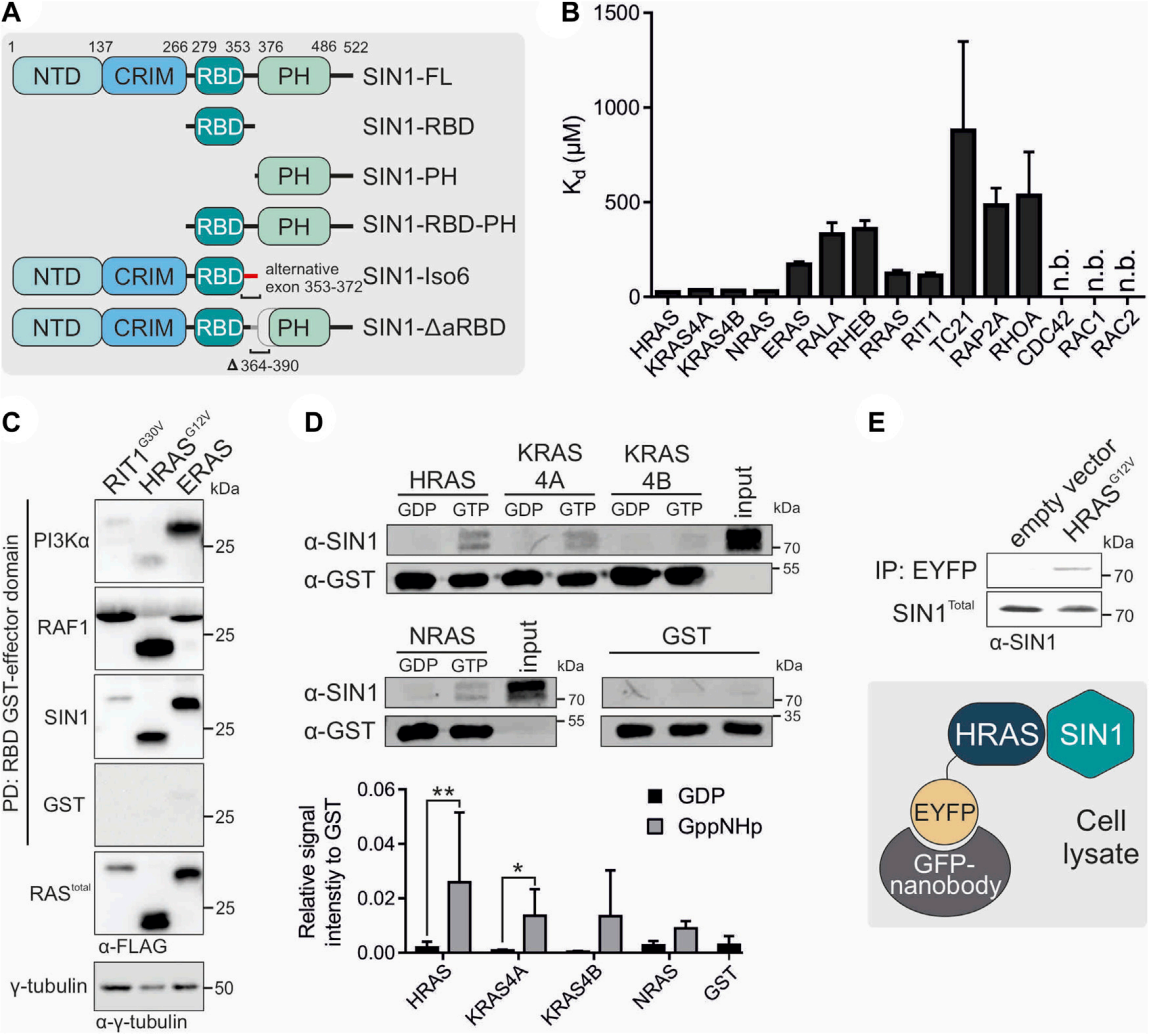
Interaction partners of SIN1. **(A)** Schematic domain organization of SIN1 and its generated fragments and variants. Numbers indicate amino acid numbering of isoform 1 (full length). **(B)** Fluorescence polarization analysis of SIN1-RBD with mGppNHp-labeled GTPases. N.b. indicates no binding observed. All K_d_ values are provided in Table 1. **(C)** Pull-down assay of the GST-bound RBDs of PI3Kα, RAF1 and SIN1 with overexpressed RIT1^G30V^, HRAS^G12V^ and ERAS containing a FLAG-Tag. γ-Tubulin served as a loading control, and GST alone served as the negative control. **(D)** Pull-down experiment with GST-bound HRAS, KRAS4A, KRAS4B or NRAS, labeled with GDP or GppNHp, and endogenous SIN1-FL in HEK293 lysates. GST alone served as a negative control. Bar charts were obtained from independent experiments. (HRAS, KRAS4A, NRAS n = 3; KRAS4B n = 2; GST n = 4). Data sets were evaluated in a two-tailed ratio paired *t* test using GraphPad Prism 6. HRAS GDP vs. GppNHp *p* = 0.0010, KRAS4A GDP vs. GppNHp *p* = 0.0114, KRAS4B and NRAS not significant. **(E)** Co-immunoprecipitation analysis of overexpressed EYFP-HRAS in COS7 cells. Proteins were immunoprecipitated using GFP nanobodies.

To characterize the binding of SIN1 to RAS, we used several fragments of SIN1, including the full length (FL), isoform 6 (Iso6), ∆aRBD, which lacks amino acids 364-390 compared to the full length of SIN1, the RBD-PH tandem, and the isolated domains RBD and PH (Figure 1A). We investigated the physical interaction of different members of the RAS superfamily with the RBD *in vitro*, checked protein binding in cell-based experiments with SIN1-FL, and identified important amino acids for interaction based on a binding model of the RBD and HRAS. Moreover, we characterized the interaction of the RBD-PH tandem with the classical RAS proteins and analyzed the membrane binding of the PH and RBD-PH proteins. We also investigated the binding of the RBD-PH domain with liposomes in the presence or absence of RAS. A cell-based study analyzed the distribution of endogenous SIN1-FL in the cytosolic and membrane fractions of HEK293 cells. We checked the phosphorylation levels of pAKT S473 after overexpressing RAS or SIN1 ∆aRBD and isoform six to monitor the activity of the mTORC2.

### 3.1 SIN1-RBD binds to all classical RAS proteins

The first aim of our study was to identify direct binding partners of SIN1-RBD (aa 279-353) within the RAS superfamily. Therefore, we investigated the binding of 15 different RAS and RHO proteins using fluorescence polarization measurements (Figure 1B; Table 1). In addition to the classical RAS proteins HRAS, NRAS, and the isoforms KRAS4A, and KRAS4B, we investigated RRAS1, RRAS2 (or TC21), ERAS, RIT1, RALA, RHEB, RAP2A, RHOA, CDC42, RAC1 and RAC2. The K_d_ values were determined *via* the titration of increasing concentrations of the SIN1-RBD to the mGppNHp-bound GTPases (Supplementary Figure S1). The classical RAS proteins exhibited the highest affinities that ranged from 24 to 35 μM, followed by RIT1, RRAS, and ERAS, with K_d_ values of 112, 123, and 170 μM, respectively (Table 1). The other tested GTPases exhibited binding affinities greater than 300 μM, which are most likely not relevant in cell signaling. Among the RHO GTPases, RHOA was the only protein that showed very weak binding above 500 µM.

To compare the binding of the SIN1-RBD to the well-known RAS effectors RAF1 and PI3Kα, we performed pull-down analyses of the three GST-fused RBDs with the hyperactive GTPases RIT1^G30V^, HRAS^G12V^ and constitutively active ERAS (Nakhaei-Rad et al., 2015). GST alone served as the negative and γ-tubulin as the loading control (Figure 1C). ERAS, which had a low affinity for SIN1-RBD *in vitro,* can bind in cells as strong as HRAS^G12V^, while RIT1^G30V^ displayed weak binding to SIN1 and PI3Kα-RBD and strong binding to RAF1-RBD. HRAS^G12V^ bound strongly to RAF1, moderately to SIN1 and weakly to PI3Kα. Notably, ERAS showed strong binding to all RBDs but the highest engagement to PI3Kα.

We examined whether binding of the SIN1-RBD was nucleotide dependent or independent and confirmed GTP-dependent binding in fluorescence polarization experiments using HRAS•mGDP vs. HRAS•mGppNHp (Supplementary Figure S2). Pull-down experiments of purified GST-fused HRAS, KRAS4A, KRAS4B and NRAS determined the binding of endogenous SIN1-FL with GDP- or GppNHp-bound RAS. The experiment clearly showed the binding of only GppNHp-bound RAS proteins (Figure 1D). The interaction of endogenous SIN1-FL with HRAS was confirmed by a co-immunoprecipitation experiment using overexpressed EYFP-HRAS^G12V^ (GAP-insensitive and therefore mostly GTP-bound mutant; Figure 1E).

### 3.2 Identification of critical SIN1/HRAS interacting residues

**FIGURE 2:**
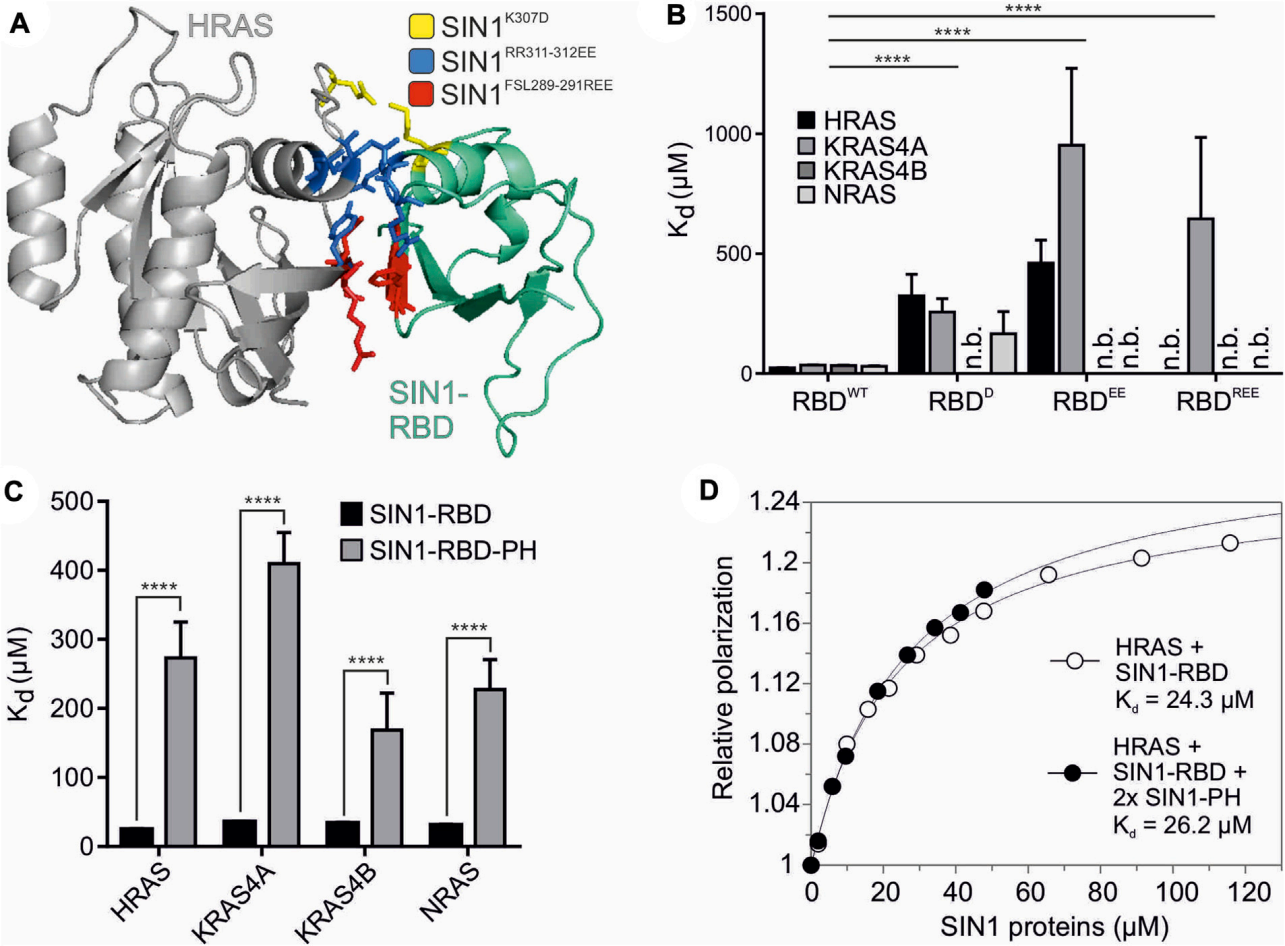
Structural analysis of SIN1-RBD and RBD-PH domains. **(A)** The interaction interface of HRAS (gray) and SIN1-RBD (teal) is highlighted in the model of their complex constructed based on the C-RAF RBD structure (PDB: 4G0N). Critical and mutated residues in the SIN1-RBD are colored as follows: SIN1^K307D^ (yellow), SIN1^RR311-312EE^ (blue), and SIN1^FSL289-291REE^ (red). **(B)** Fluorescence polarization analysis of the RBD mutants compared to the WT SIN1-RBD with the mGppNHp-labeled classical RAS proteins HRAS, KRAS4A, KRAS4B, and NRAS. All K_d_ values are shown in Table 2. The difference in the binding affinity of WT SIN1-RBD in comparison to the three SIN1-RBD mutants was highly significant for all proteins (two-tailed unpaired *t* test, *p* < 0.0001). **(C)** Fluorescence polarization analysis of the SIN1-RBD-PH tandem construct with mGppNHp-labeled classical RAS proteins compared to SIN1-RBD binding alone. All K_d_ values are provided in Table 2. **(D)** Fluorescence polarization graphs of HRAS mGppNHp with SIN1-RBD (K_d_ = 24 ± 2 µM) and double the amount of SIN1-PH (K_d_ = 27 ± 2 µM). SIN1-RBD and SIN1-PH were premixed and pre-incubated before titration.

To identify potential contact sites of SIN1-RBD on RAS, the SIN1-RBD structure in complex with HRAS was modeled based on sequence homology to the complex of RAF1-RBD with GppNHp-bound HRAS (PDB: 4G0N). We analyzed the interaction interface between HRAS and SIN1-RBD and selected several SIN1 residues in close proximity to HRAS that may be responsible for the direct interaction between RAS proteins and SIN1 (Figure 2A). Based on these identified interacting residues, three different mutants of SIN1-RBD were designed (Figure 2A): SIN1-RBD^K307D^, SIN1-RBD^RR311-312EE^, and SIN1-RBD^FSL289-291REE^. Residues were substituted with amino acids with opposite charges to generate repulsion between the interacting residues.

The biophysical measurements revealed decreased binding of SIN1-RBD mutants with mGppNHp-bound HRAS, KRAS4A, KRAS4B and NRAS (Figure 2B and Supplementary Figure S3). The K_d_ of the single mutant was 5- to 15-fold higher than SIN1-RBD^WT^ (Tables 1 and 2). The double and triple mutants further decreased the binding affinity. All mutations abolished SIN1-RBD binding capability to KRAS4B but were still bound to KRAS4A with a low affinity. The recently published structure of KRAS binding with SIN1-RBD by Castel *et al.* (PDB: 7LC1 and 7LC2) and Zheng *et al.* (PDB: 7VVB) and HRAS binding of SIN1-RBD by Zheng et al. (PDB: 7VV9) confirmed that these residues are in close proximity to the switch I region of KRAS and are very likely involved in a physical interaction (Supplementary Figure S4) (Castel et al., 2021; Zheng et al., 2022). Notably, our SIN1 mutations were generated and characterized far before these structures of the SIN1-RAS complexes were published.

**TABLE 2:** The Interaction of SIN1-RBD mutants with RAS proteins.

Protein	HRAS	KRAS4A	KRAS4B	NRAS
SIN-RBD^K307D^	324 ± 90	256 ± 56	No binding observed	166 ± 93
SIN-RBD ^RR311,312EE^	461 ± 95	952 ± 321	No binding observed	No binding observed
SIN-RBD ^FSL289-291REE^	No binding observed	654 ± 339	No binding observed	No binding observed
SIN1-RBD-PH	273 ± 52	410 ± 45	168 ± 54	227 ± 43

Values displayed are K_d_ ± SD, in µM.

### 3.3 SIN1-RBD-PH tandem domain has much lower binding to RAS than RBD alone

We investigated the interaction of the tandem SIN1-RBD-PH domain with classical RAS proteins (HRAS, KRAS4A, KRAS4B and NRAS) using fluorescence polarization (Figure 2C and Supplementary Figure S5). Obtained K_d_ values were 5- to 10-fold higher than the SIN1-RBD interaction (Table 2), which strongly suggests a possible intermolecular interaction between the PH and RBD domains. To examine whether this RBD-PH interaction is due to direct binding of the individual domains or occurs only in the linked tandem domain, fluorescence polarization measurement of SIN1-RBD with HRAS•mGppNHp in the presence of 2x excess SIN1-PH was performed and resulted in a K_d_ of 27 ± 2 μM, which was similar to the K_d_ obtained for SIN1-RBD alone (24 ± 2 µM) (Figure 2D). The SIN1-PH domain alone showed no binding to HRAS (Supplementary Figure S6).

### 3.4 SIN1-PH and RBD-PH associate with the membrane

**FIGURE 3:**
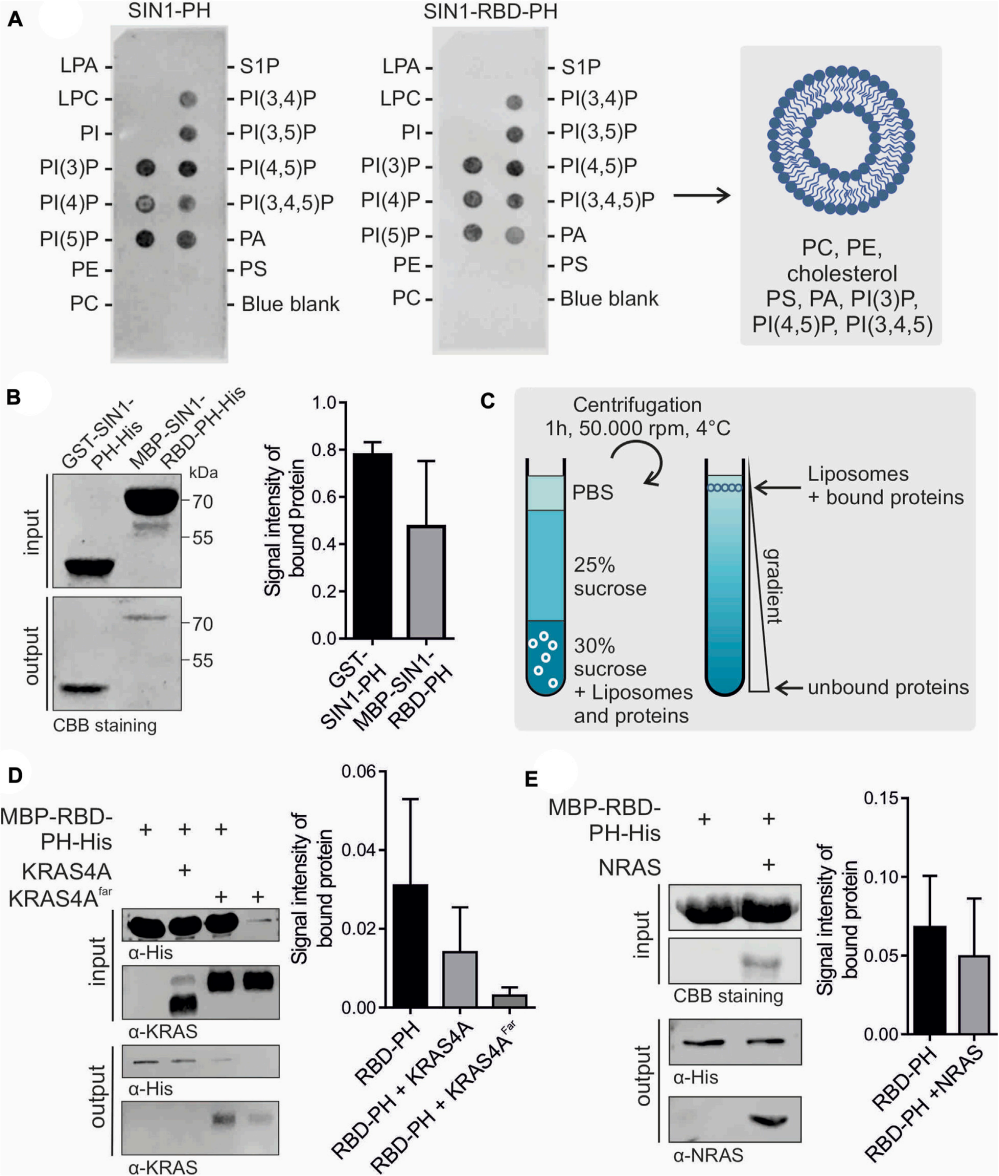
Membrane association of SIN1-PH and RBD-PH. **(A)** PIP-Strip assay of SIN1-PH (left) and SIN1-RBD-PH (right). The blue blank served as a negative control. CBB stands for Coomassie brilliant blue. **(B)** Flotation assay of SIN1-PH and SIN1-RBD-PH domains stained with Coomassie. (n = 3) Not significant. **(C)** Schematic principle of a liposome flotation assay using a sucrose gradient and ultracentrifugation. **(D)** Flotation assay of SIN1-RBD-PH in the presence of KRAS4A purified from *E. coli* and KRAS4A^farnesylated^ from insect cells (n = 3). **(E)** Flotation assay of SIN1-RBD-PH with NRAS purified from *E. coli.* (n = 3). Data sets were evaluated in a two-tailed unpaired *t* test using GraphPad Prism six and displayed no significance. CBB stands for Coomassie brilliant blue.

We further focused on the investigation of the membrane binding ability and lipid selectivity of SIN1-PH and SIN1-RBD-PH domains using PIP-Strips (Figure 3A), liposome sedimentation (Supplementary Figure S7), and liposome flotation assays (Figure 3B). PIP-Strip assays confirmed a similar selectivity and comparable intensity of the PH and RBD-PH domains toward all phosphoinositides. The strongest binding was detected for PI(3)P, PI(5)P, and PI(4,5)P. PH and RBD-PH bound to phosphatidic acid (PA) but no other lipids. Based on this assay, we used liposomes containing PC, PE, PS, PA, PI(3)P, PI(4,5)P, PI(3,4,5)P, and cholesterol for subsequent experiments to cover all possible binding modes.

We confirmed the binding of the GST-SIN1-PH domain to our synthetic liposomes compared to the GST control in a liposome sedimentation assay (Supplementary Figure S7). Most protein was detected in the liposome/pellet fraction. The GST control was only detectable in the supernatant. We checked the membrane binding ability of the MBP-SIN1-RBD-PH domain in a liposome flotation assay. Proteins were mixed and incubated with fluorescent-labeled synthetic liposomes and stacked in a glucose gradient. After ultracentrifugation, liposomes, including bound proteins, were isolated and detected using Coomassie staining or Western blotting (Figures 3B,C). As a negative control, we used only purified MBP. In addition, we checked the lipid selectivity of the SIN1-PH domain by using negative liposomes containing only 90% PC and 10% PE, which showed no liposome association in the flotation assay (Supplementary Figure S8). Our results showed the binding of SIN1-PH and RBD-PH to the liposomes, with the binding of the latter being comparably weaker. This effect is most likely caused by interdomain interaction between the RBD and the PH domain discussed in Section 3.3. A remaining question is whether RAS binding to the RBD is supported by PH domain binding to the membrane or whether this PH-membrane interaction is regulated by RAS.

### 3.5 RAS weakens the membrane interaction of SIN1-RBD-PH

To determine the effect of RAS on the membrane binding of SIN1-RBD-PH, we performed liposome flotation assays using GppNHp-bound KRAS4A without and with posttranslational modifications, such as farnesylation in its CAAX box, that facilitates its binding to the membrane. The results indicated weaker binding of RBD-PH to the liposomes in the presence of non-farnesylated and farnesylated KRAS4A (Figure 3D) as well as in the presence of GppNHp-bound NRAS (Figure 3E). Both results suggest an influence of RAS on the localization of SIN1 and the mTORC2 complex within the cell.

### 3.6 SIN1-FL is always partially membrane associated

**FIGURE 4:**
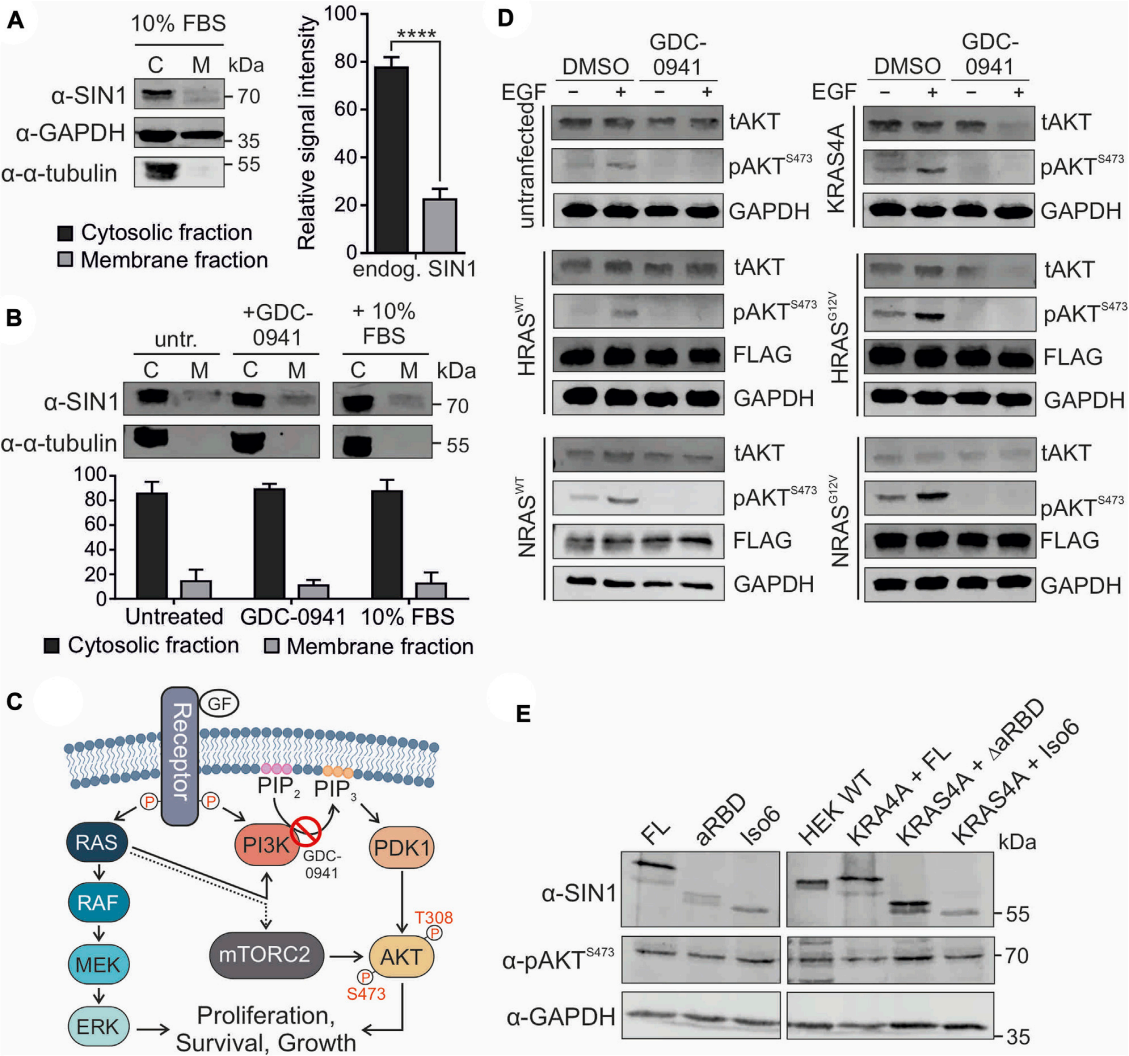
Localization and signaling of SIN1. **(A)** Partial fractionation of HEK293 cells into cytosolic and membrane fractions (n = 6, two-tailed paired *t* test *p* < 0.0001). GAPDH served as a loading control and α-tubulin as a control for the cytosolic fraction. **(B)** Partial fractionation of 16 h serum staved HEK293 cells under untreated, 1 µM GDC-0941 inhibited or 10% FBS-stimulated conditions (1 h each) (not significant, two-tailed unpaired *t* test). α-Tubulin served as a control for the cytoplasmic fraction (98:2). **(C)** Illustration of the RAS-MAPK and PI3K-AKT pathways induced by EGF. The dotted line indicates an unknown effect of RAS on the mTORC2 complex. **(D)** Stimulation experiment with untransfected or EYFP-KRAS4A-, HRAS^WT−^, HRAS^G12V−^, NRAS^WT−^ or NRAS^G12V^-transfected HEK293 cells. For KRAS4A, green fluorescence was used as an expression control. The other constructs carried an N-terminal FLAG-tag and were detected using an α-FLAG antibody. GAPDH served as a loading control. tAKT and pAKT^S473^ levels were estimated after serum starvation, followed by treatment with DMSO or 1 µM GDC-0941 for 1 h and stimulation with 100 ng/ml EGF for 20 min as indicated. **(E)** Western blot analysis of overexpressed SIN1 constructs (FL, ∆aRBD, and Iso6) or co-expressed SIN1 variants with EYFP-KRAS4A in HEK293-SIN1 knock-out cells, clone 2A, under normal culture conditions (10% FBS). For KRAS4A, green fluorescence was used as an expression control. SIN1 antibody indicates the overexpressed variants and indicates knock-out compared to wild-type (WT) cells. The pAKTS473 levels were analyzed with a specific antibody. GAPDH served as a loading control.

Different localizations of SIN1 within the cell have been reported in the past few years. We have now applied different approaches to study the translocation of endogenous SIN1 to the membrane. A first approach was to partially fractionate HEK293 cells and determine the ratio between cytosolic SIN1 and membrane fractions. Data from six independent experiments showed that most of the endogenous SIN1-FL protein was present in the cytosolic fraction, with a ratio of approximately 77:23 (*p* ≤ 0.0001; Figure 4A). In a next step, we examined the effects of the PI3K-AKT pathway on the localization of SIN1 in the membrane. We used two opposing conditions, either inhibiting the pathway with GDC-0941, a small molecule PI3K inhibitor, or stimulating it with 10% FBS. The results presented in Figure 4B did not lead to an obvious shift of SIN1 between the cytosolic and membrane fractions compared to the serum-starved cells. Of note, the ratio of cytosolic to membrane immunodetectable SIN1 was quite similar among the three conditions (serum-starved 86:14; GDC-0941 treated 89:11; FBS stimulation 88:12). As a control for the cytosolic fraction, we used α-tubulin, which was present in the latter at approximately 96-98%, indicating only a very weak contamination of approximately 2-4% of the cytosolic fraction in the membrane fraction. The membrane fraction was checked using Na^+^/K^+^-ATPase as a marker protein (Supplementary Figure S9).

### 3.7 RAS overexpression does not alter AKT S473 phosphorylation

The RAS signaling pathway follows two canonical routes: one via RAF and MEK toward ERK, the other via PI3K activation toward AKT phosphorylation at T308 (Muñoz-Maldonado et al., 2019). As described before, the phosphorylation of AKT at S473 mostly depends on the mTORC2 complex and serves as a readout for its activity (Sarbassov et al., 2005; Castel et al., 2021). The PI3K inhibitor GDC-0941 blocks the conversion of PIP_2_ to PIP_3_ and many translocation events of PIP_3_-dependent PH domain-containing proteins, such as AKT (Figure 4C). To investigate the influence of RAS on the phosphorylation of AKT, we overexpressed wild-type KRAS4A, HRAS, NRAS, and the hyperactive variants HRAS^G12V^ and NRAS^G12V^ in HEK293 cells (Figure 4D). Cells were serum starved and treated as indicated with EGF and/or GDC-0941. Stimulation with EGF led to strong AKT^S473^ phosphorylation in all cases (Figure 4D). The G12V mutation further promoted signaling, which was likely due to the constant activation of the PI3K pathway. The GDC-0941 inhibitor completely abolished AKT^S473^ phosphorylation. This could be further supported by stimulating HEK293 cells with EGF, insulin and 10% FBS in combination with the GDC-0941 inhibitor, which abolished the phosphorylation of AKT S473 in all cases (Supplementary Figure S10). This experiment strongly supports the need for PI3K activity for AKT phosphorylation. In order to better understand the role of the PH domain and eventually of the aRBD, as suggested by Castel *et al.*, on the activity of the mTORC2 complex toward AKT we overexpressed several SIN1 variants. SIN1-∆aRBD lacks amino acids 364-390, and isoform 6 (Iso6) is missing the whole PH domain and contains an alternative exon 9a instead of the aRBD. Because endogenous SIN1 could interfere with the effect of transfected SIN1 variants, which could be caused by the formation of heterodimers (Stuttfeld et al., 2018; Scaiola et al., 2020), we performed a CRISPR/Cas9 knock-out of SIN1 in HEK293 cells and selected a single clone (clone 2A) for further overexpression experiments. The single clone showed no signal for either SIN1 antibody or phosphorylated AKT at S473 (Supplementary Figure S11). All overexpressed SIN1 proteins were able to rescue phosphorylation of AKTS473 and did not dramatically increase or decrease AKT phosphorylation when co-expressed with KRAS4A (Figure 4E).

Overall, our cell biological results suggest PI3K-dependent phosphorylation of AKT^S473^, likely through recruitment of AKT to the membrane, but not through alteration of SIN1 and thus mTORC2 localization within the cell, as a small fraction appears to be constantly localized to the membrane. In addition, we failed to demonstrate a RAS-dependent increase in AKT-S473 phosphorylation after GDC-0941 treatment, as well as the ability of SIN1-FL, ∆aRBD, and isoform six to rescue pAKTS473 levels in SIN1 knockout cells. It seems that some specific issues related to AKT regulation and feedback mechanisms still need to be clarified.

## 4 Discussion

The role of the SIN1-RBD and the interaction of RAS and SIN1 raised more questions than answers during the past years. Zheng et al. (Zheng et al., 2022), Castel et al. (Castel et al., 2021) and Liu et al. (Liu et al., 2015) added new interesting concepts for the function of the RBD and the PH-domain of SIN1 in the complex regulatory network of mTORC2. Our study adds the influence of RAS on the membrane binding of SIN1 as another functional factor.

Schroder *et al.* (2007) described the RAS binding domain of SIN1 and showed the association of HRAS^G12V^ and KRAS4B^G12V^ with SIN1 (Schroder et al., 2007). Castel *et al.* revised this study and introduced KRAS4A•GTP as the ultimate binding partner for SIN1. Consistent with Zheng *et al.*, who showed an association with HRAS, KRAS, and NRAS, we identified the four classical RAS proteins (HRAS, KRAS4A, KRAS4B, and NRAS) as the strongest binders of SIN1-RBD and confirmed the GTP-dependent binding of these proteins with SIN1-FL in cells. We have added ERAS, RRAS, and RIT1 to the list of potential binding partners based on fluorescence polarization which serves as a sensitive biophysical method for the identification of protein complexes with lower binding affinities. RIT1-SIN1 interaction has also been shown previously to be required for oxidative stress survival (Cai, Andres and Reiner, 2014). Pull-down assays confirmed the binding of ERAS and RIT1 with SIN1-RBD and further confirmed the preferable binding of HRAS^G12V^ with RAF1, SIN1, and PI3Kα RBDs, which is exactly the order shown by Zheng *et al.*

Our structural analysis identified a few residues for the interaction of SIN1-RBD with the switch region of HRAS. The residues R311, R312, F289, S290 and L291, which showed a much lower or complete loss of binding after mutation, were also identified by Castel *et al.* and Zheng *et al.*, and collectively highlight these residues as the main interaction sites of SIN1 and RAS, although this binding site may not be exclusive (Castel et al., 2021; Zheng et al., 2022). Interestingly, the F289L, S290F, and R311Q mutations were found to be SIN1 cancer mutations (COSMIC database, 2022), suggesting their critical role in SIN1 function. The RBD-PH construct showed much weaker binding to RAS than RBD alone, which was also reported by Castel *et al.*, Zheng *et al.*, and by this study. Contrary to these results, we did not detect a direct interaction of the free PH domain with the RBD but proposed a low-affinity RBD-PH tandem interdomain interaction.

The PH domain of SIN1 was examined by Liu *et al.* (2015), who reported the binding of the PH domain to mTOR in an inhibitory manner and to PIP_3_ to activate the complex by opening the mTORC2 binding pocket. Their study concluded that the activity of the mTORC2 complex was PI3K dependent, which produced PIP_3_ in response to growth factors. Ebner *et al.* (2017) investigated the localization and activity of the mTORC2 complex in the cell using a new reporter system called LocaTOR2 (localization of mTOR complex 2), based on its effector AKT2. The study identified different pools of the mTORC2 complex at the plasma membrane, mitochondria, and endosomal vesicles. This finding highlighted that mTORC2 activity at the plasma membrane was PI3K independent and activated the reporter upon PI3K inhibition with GDC-0941. Nevertheless, the substrate AKT was not recruited to the PM under PI3K inhibition, which indicates that the phosphorylation of AKT is PI3K dependent based on its localization, but mTORC2 activity does not need PI3K for its activity. Our study also shows stimulation-independent SIN1 localization at the plasma membrane, which was supported by the binding of the SIN1-PH domain to phosphoinositides other than only PIP_3_. Other members of the mTORC2 complex may also trigger membrane localization. The domain organization of RICTOR is not completely defined, and two possible PH domains (including one split PH domain), in addition to HEAT and WD repeats, were identified based on sequence and structural similarities (Zhou et al., 2015).

**FIGURE 5:**
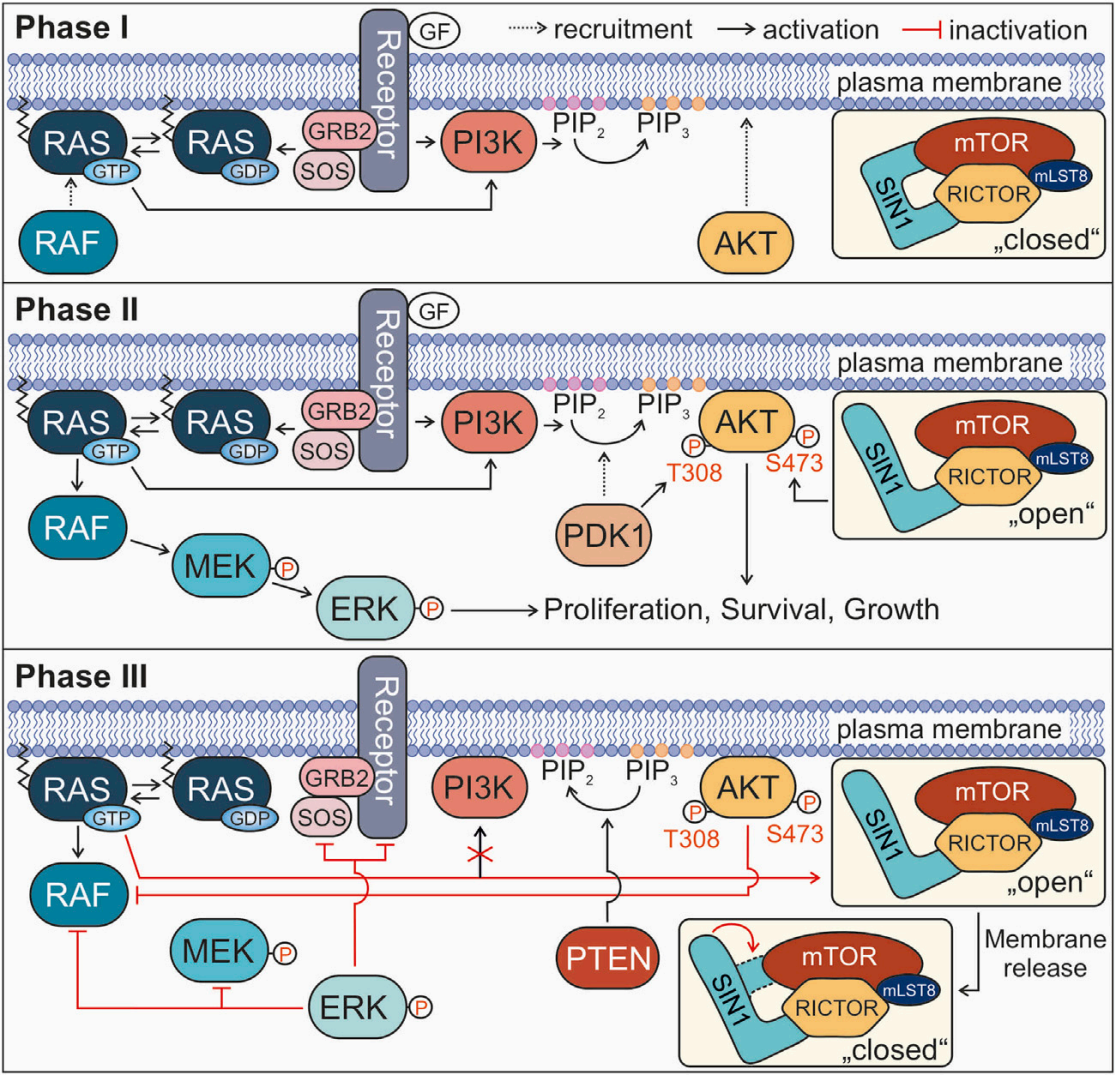
Proposed model for the involvement of the RAS-SIN1 interaction in signal termination. Phases I, II, and III describe the signaling process from growth factor binding to signal termination. For more details see text.

Taken together, our study shows for the first time the membrane association of SIN1-RBD-PH compared to the PH domain alone and additionally analyzed this interaction in the presence of farnesylated and non-farnesylated RAS. We showed that RAS interfered with the binding of SIN1-RBD-PH to liposomes. Mechanistically, it is tempting to hypothesize that RAS association with the membrane-bound SIN1 results in spatial rearrangement of the RBD-PH tandem followed by SIN1 dissociation from the membrane and then subsequently from RAS. The RAS-SIN1 interaction is consequently accompanied by crosstalk and feedback mechanisms of the RAS-MAPK and PI3K-AKT signaling pathways (Figure 5). The binding of RAS to SIN1 rather than PI3K may reduce the activity of the PI3K-AKT pathway and AKT phosphorylation by mTORC2, which is alternatively followed by the disassembly of the mTORC2 complex. This assumption is based on our observation that some mTORC2 continuously resides at the membrane. It suggests that the spatiotemporal control of AKT, its recruitment and clustering to lipid rafts is the key to switching the AKT signaling pathway on and off.

All in all, the results of the present study and the previous work by other groups led to the following model proposing the role of SIN1-RAS interaction in the negative feedback loop of RAS-RAF-MEK-ERK and PI3K-AKT signaling pathways. Our model divides the activation of RAS and its downstream effectors into three phases until the termination of signal transduction (Figure 5). In phase I (the initiation phase), RAS activation through a GDP/GTP exchange by the RTK-GRB2-SOS axis (Kazemein Jasemi and Reza Ahmadian, 2022) transmits the extracellular signals (*e.g.*, EGF) towards both RAF and PI3K. This recruits RAF to the plasma membrane, and activates PI3K to catalyze the conversion of PIP_2_ to PIP_3_, followed by membrane recruitment of AKT. In this phase, the mTORC2 complex with SIN1 is present in a partially “closed” conformation at the membrane and therefore, inaccessible for RAS. The inhibitory binding of the PH domain to mTOR blocks the catalytic binding pocket of the complex (Liu et al., 2015). In phase II (maximum signaling phase) the two canonical RAS signaling pathways are fully activated. RAF is activated by several dephosphorylation, conformational change and homo- or heterodimerization events and transmits the signal to MEK and ERK (Lavoie and Therrien, 2022). PDK1 recruitment to PIP_3_-rich clusters results in T308 phosphorylation and activation of AKT. At the same time, SIN1 switches into an open conformation, which may be triggered by the association of the PH domain with the membrane accompanied with mTORC2 substrate recognition, which seems to be different for AKT and SGK1 (Yu et al., 2022). The mTORC2 complex phosphorylates AKT at S473, leading to its complete activation. Both phosphorylated ERK and AKT now stimulate cell responses, such as proliferation, survival and cell growth. In the following phase III (the signal termination), several feedback loops lead to the shutdown of the signaling processes, including: 1) RAS•GTP binding to SIN1 in its open conformation, dissociating the SIN1-PH domain from the membrane back to its closed conformation, disrupting the positive feedback loop to PI3K and interfering with the activation of AKT by mTORC2; 2) activated ERK inhibits its own signaling cascade by phosphorylating RTKs, SOS, RAF and MEK; 3) activated AKT re-phosphorylates RAF at S259 (CRAF numbering), the critical inhibitory phosphorylation site (Zimmermann and Moelling, 1999; Dhillon et al., 2002; Lake, Corrêa and Müller, 2016); 4) PTEN dephosphorylates PIP_3_ to PIP_2_ (Lee, Chen and Pandolfi, 2018). Notably, there are more negative feedback processes known, like the ubiquitination and internalization of receptors (Tomas, Futter and Eden, 2014), the inactivation of RAS by GAPs (Lorenzo and McCormick, 2020), and the negative feedback of ERK towards other signaling proteins, *e.g.*, sprouty or FRS2α (Lake, Corrêa and Müller, 2016), that were not included in the model.

## Data Availability

The original contributions presented in the study are included in the article/Supplementary Material, further inquiries can be directed to the corresponding author.
