# Supplementary material for: New mechanistic insights into the RAS-SIN1 interaction at the membrane
Source: Front Cell Dev Biol. 2022 Oct 6;10:987754. doi: 10.3389/fcell.2022.987754 (PMC9583166; doi:10.3389/fcell.2022.987754)
Supplement: Supplementary file 1 [file DataSheet1.pdf]

Supplementary Material

New Insights into the RAS-SIN1 interaction at the membrane

Silke Pudewell, Jana Lissy, Hossein Nakhaeizadeh, Niloufar Mosaddeghzadeh, Saeideh Nakhaei-Rad, Radovan Dvorsky, Mohammad R. Ahmadian*

Institute of Biochemistry and Molecular Biology II, Medical Faculty, Heinrich-Heine University, 40225 Düsseldorf, Germany

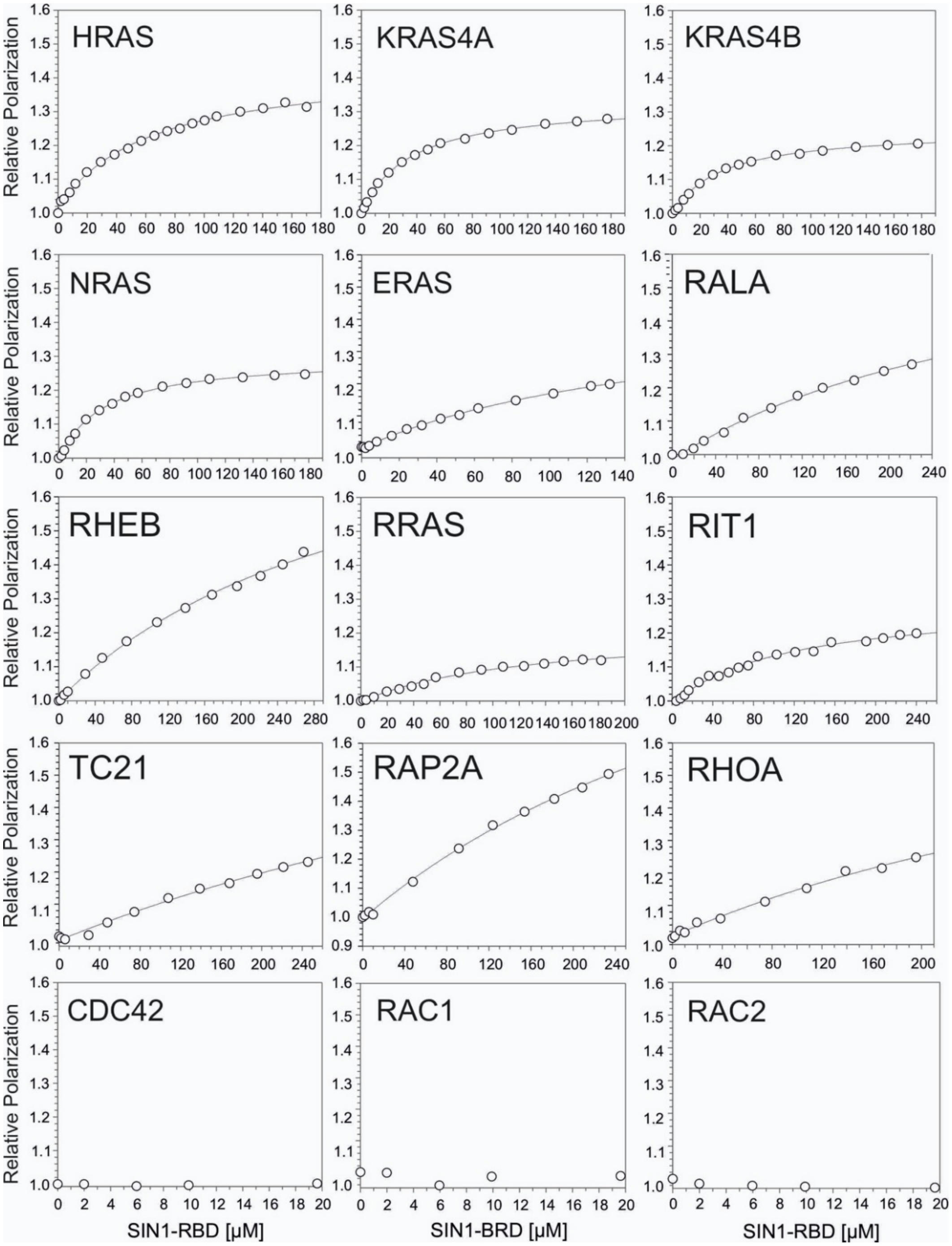

Supplementary Figure S1. Fluorescence polarization measurement of the interaction of SIN1-RBD with different RAS and RHO GTPases. mGppNHp-bound GTPases were titrated with increasing concentrations of MBP-SIN1-RBD. K_d values were obtained as described in Material and Methods and collectively presented in Table 1, and illustrated as bar charts in Figure 1B. The data points collected for the calulation of the K_d values were: HRAS n=20; KRAS4A n=16; KRAS4B n=16; NRAS n=16; ERAS n=15; RALA n=12; RHEB n=14; RRAS n=17; RIT1 n=19; TC21 n=12; RAP2A n=11; RHOA n=11; CDC42 n=5; RAC1 n=5; RAC2 n=5.

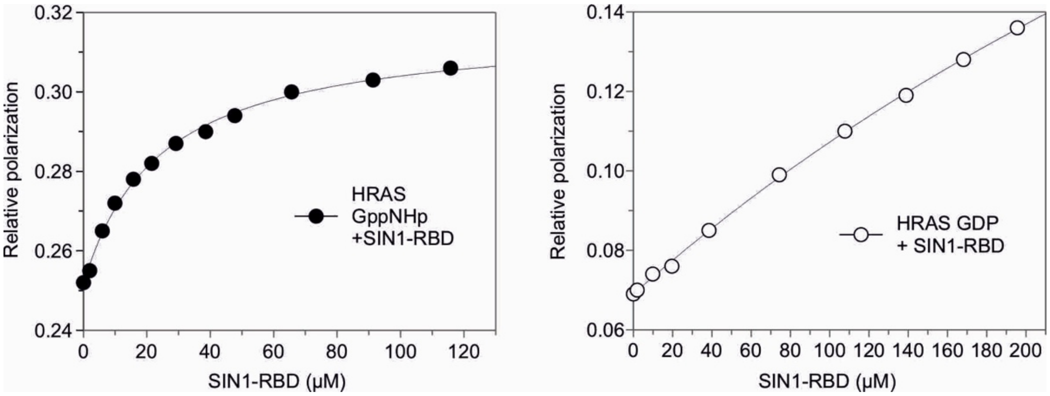

Supplementary Figure S2. Fluorescence polarization measurement of the interaction of SIN1-RBD with mGppNHp-bound and mGDP-bound HRAS. SIN1-HRAS interaction was measured using fluorescence polarization by titrating increasing concentrations of SIN1-RBD to mGppNHp-bound HRAS (left panel; K_d = 24.3 ± 1.7) and mGDP-bound HRAS (right panel; K_d = 720 ± 94 µM), respectively. The data clearly indicates the GTP-dependent binding of SIN1-RBD. The data points collected for the calulation of the K_d values for the interaction of HRAS•mGDP with SIN1-RBD was n=9.

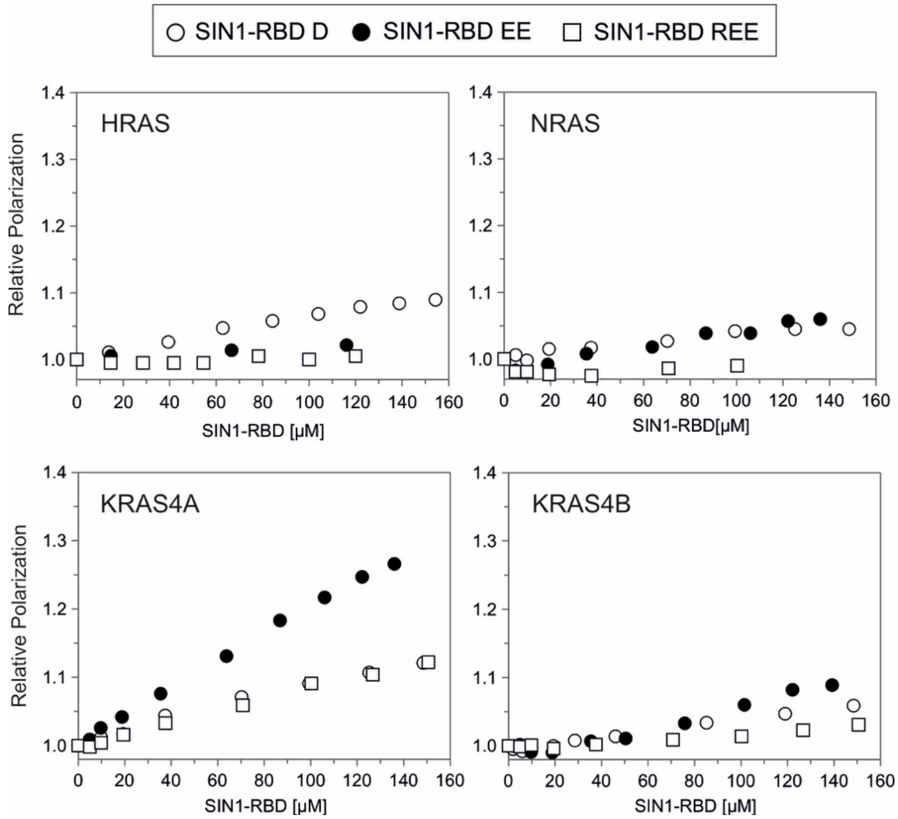

Supplementary Figure S3. Fluorescence polarization measurement of the interaction of SIN1-RBD mutants with HRAS, KRAS4A, KRAS4B and NRAS. mGppNHp-bound RAS proteins were titrated with increasing concentration of SIN1-RBD K307D, RR311-312EE or FSL289-291 mutants. K_d values were obtained as described in Material and Methods and collectively presented in Table 2 and illustrated as bar charts in Figure 2B. The data points collected for the calulation of the K_d values were: HRAS: RBD^{D} n=9, RBD^{EE} n=4, RBD^{REE} n=8; NRAS: RBD^{D} n=9, RBD^{EE} n=9, RBD^{REE} n=7; KRAS4A: RBD^{D} n=10, RBD^{EE} n=9, RBD^{REE} n=9; KRAS4B: RBD^{D} n=9, RBD^{EE} n=10, RBD^{REE} n=9.

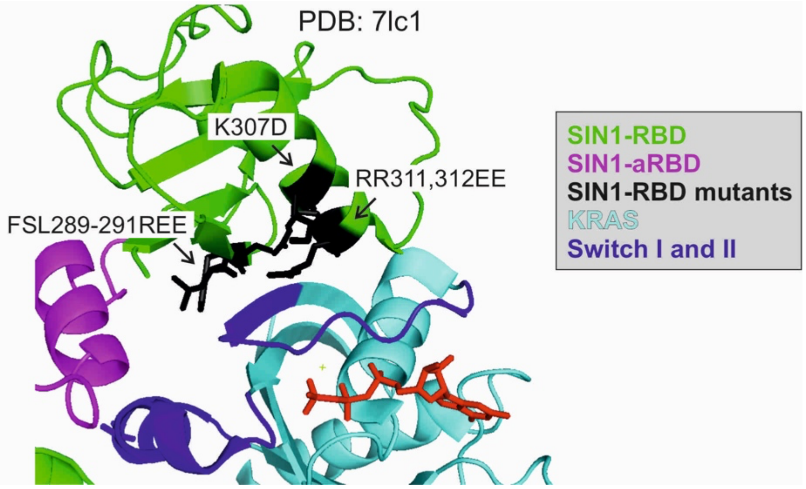

Supplementary Figure S4. Crystal structure of SIN1-RBD complex with KRAS•Gpp(CH_2)p. The structure was published by Castel *et al.* PNAS 2021 Vol. 118 No. 33. Upper structure displays SIN1-RBD (green) with the black residues indicate the amino acids that were mutated in this study based on our own modelled structure, which was generated three years before the Castel et al. was published. The pink α-helix was defined as the alternative RBD (aRBD) by Castel *et al*. (2021). The cyan structure is KRAS without the HVR, including the dark blue residues as the switch I and switch II region. The image was created with PyMOL Molecular Graphics System. The mutated (black) residues show a close proximity to the switch region I region of KRAS.

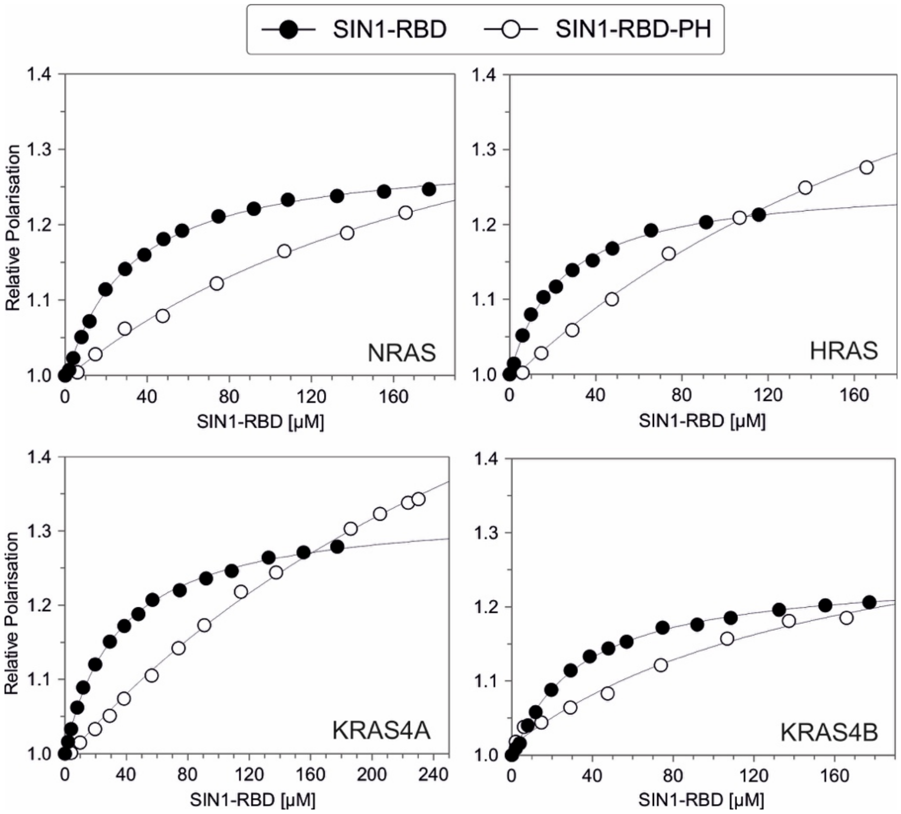

Supplementary Figure S5. Fluorescence polarization measurement of the interaction of SIN1-RBD-PH with HRAS, KRAS4A, KRAS4B and NRAS. mGppNHp-bound RAS proteins were titrated with increasing concentration of SIN1-RBD-PH. K_d values were obtained as described in Material and Methods and collectively presented in Table 2 and illustrated as bar charts in Figure 2C. The data points collected for the calulation of the K_d values were: NRAS n=8; HRAS n=8; KRAS4A n=14; KRAS4B n=10.

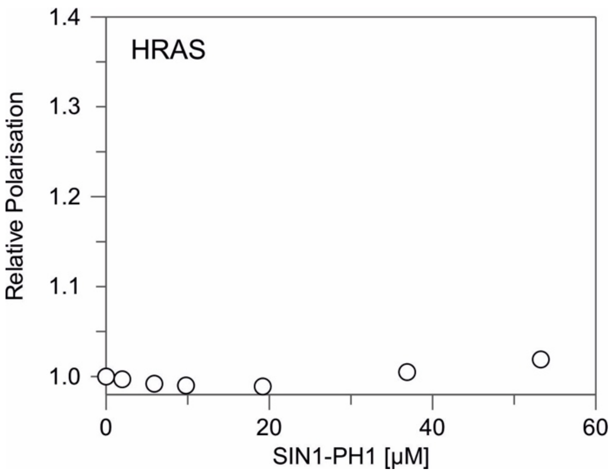

Supplementary Figure S6. Fluorescence polarization measurement of the interaction of SIN1-PH with mGppNHp-bound HRAS. The SIN1-PH domain was titrated to fluorescent labelled HRAS protein. The measurement displayed no change in polarization and indicates no binding of the PH domain towards HRAS.

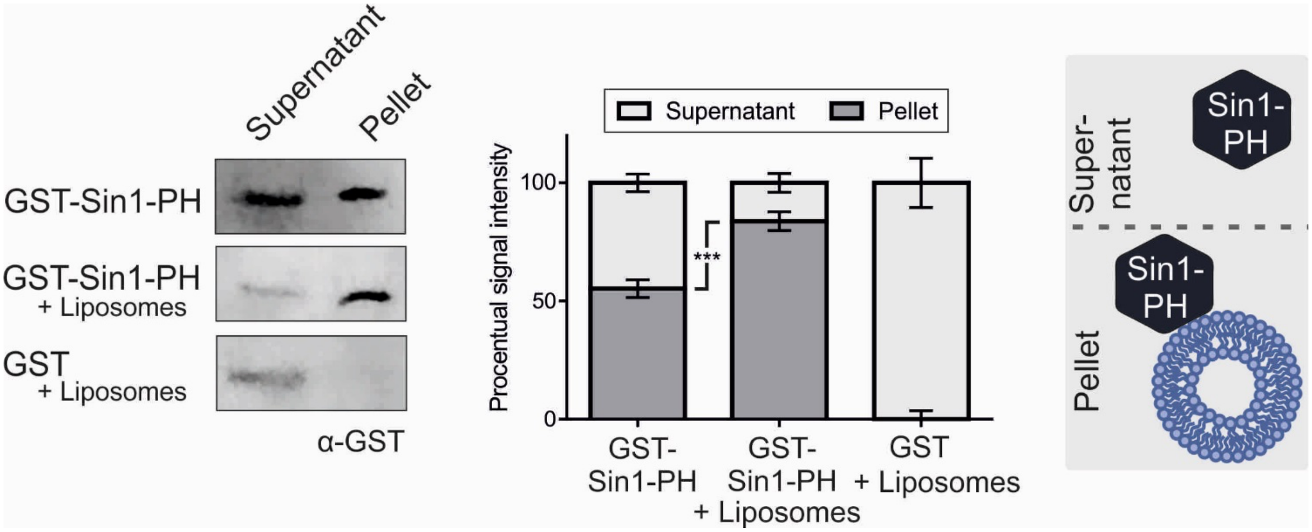

Supplementary Figure S7. Liposome sedimentation of SIN1-PH domain. Liposome sedimentation was performed as described in Material and Methods and is illustrated in the right panel. GST-SIN1-PH domain obtained a 45:55 supernatant:pellet distribution without liposomes and shifted towards the pellet fraction with 85% after adding liposomes (***, $p = 0.0005$, two-tailed, unpaired t test). GST served as a negative control and could be found 100% in the supernatant. The samples were detected with an anti-GST antibody.

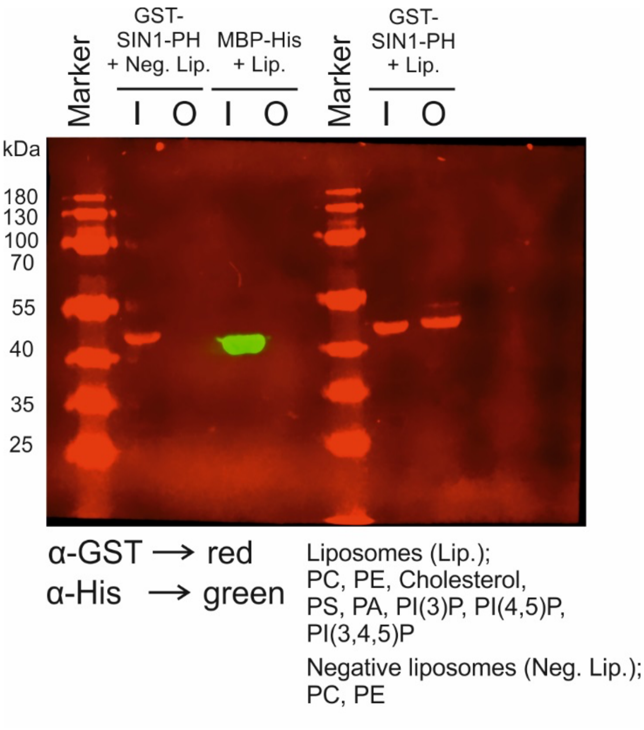

Supplementary Figure S8. Flotation assay control. The flotation assay was performed with liposomes (Lip.) containing PC, PE, Cholesterol, PS, PA, PI(3)P, PI(4,5)P and PI(3,4,5)P (concentrations as indicated in Material and Methods) for the MBP-His protein and the positive control with GST-SIN1-PH or negative liposomes (Neg. Lip.) that only contain PC (90%) and PE (10%). "I" indicate the input and "O" the output. The GST-SIN1-PH domain could be detected in the output of the liposomes containing all lipids but not in the fraction of the negative liposomes. The MBP-His was only found in the input, but not associated with the liposomes and therefore not observed in the output.

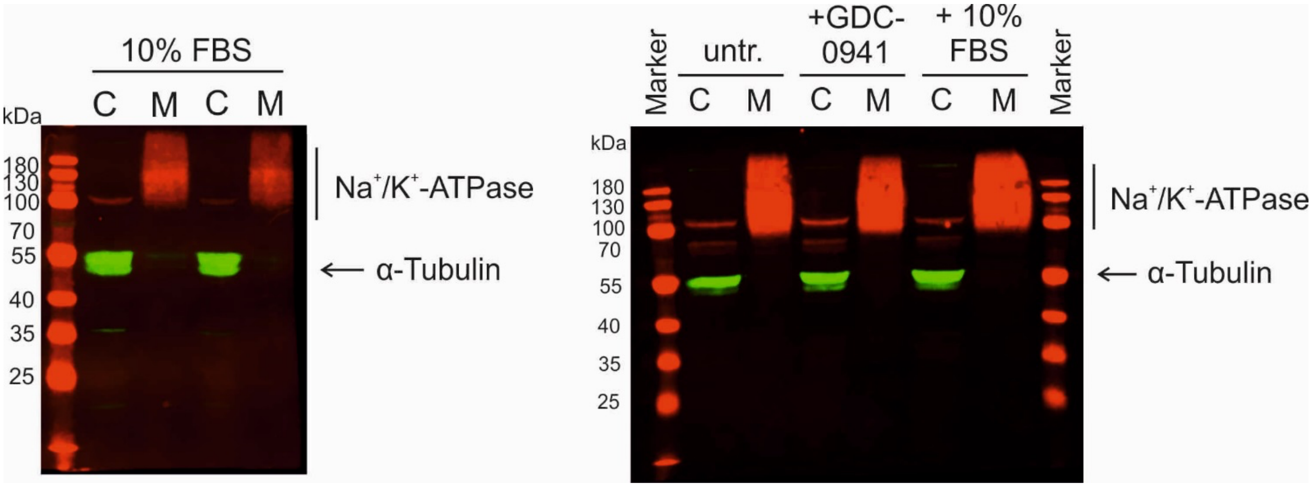

Supplementary Figure S9. Partial fractionation control. The partial fractionation was controlled by specific marker proteins. Alpha-Tubulin (green) was used for the cytosolic fraction (C) and the sodium-potassium ATPase (Na^+/K^+-ATPase) (red) for the membrane fraction (M). This control was accessed for figure 4A (left panel) and figure 4B (right panel).

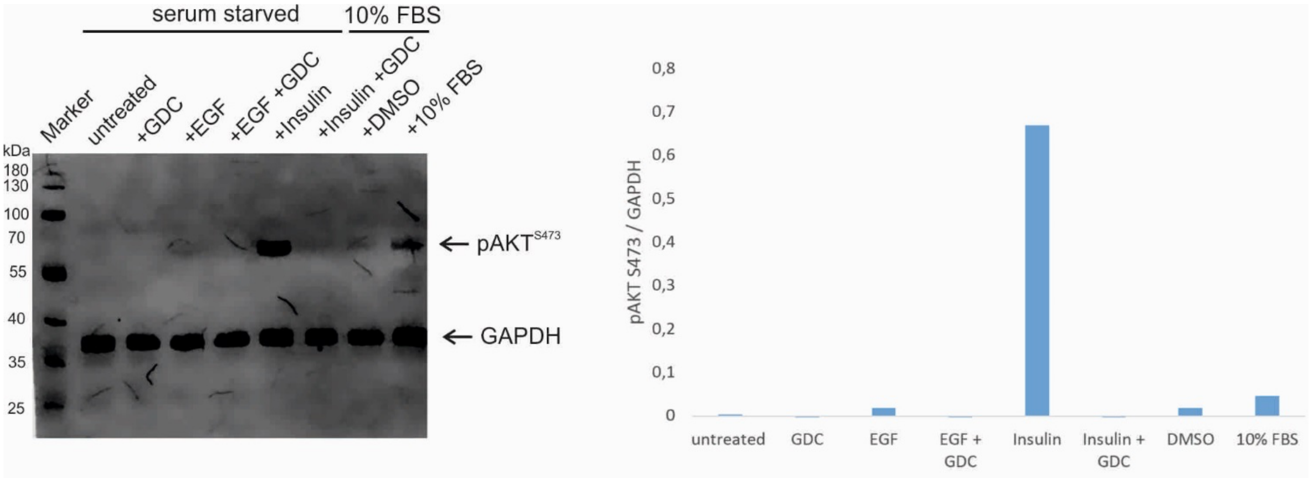

Supplementary Figure S11. Stimulation of HEK293 cells. HEK293 wild type cells were stimulated with 100 ng/ml EGF and 500 ng/ml insulin or 10% FBS and treated with or without 1µM of the PI3K inhibitor GDC-0941 (GDC) as indicated. Signal for $pAKT^{S473}$ could only be detected for the stimulated samples but not for samples simultaneously treated with GDC-0941. GAPDH served as a loading control. Quantifications of pAKT relatively to GAPDH are provided in the lower graph.

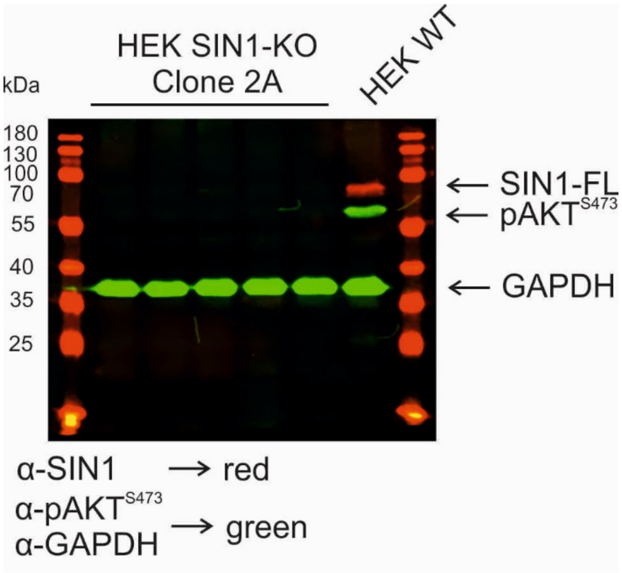

Supplementary Figure S11. Validation of the SIN1 knock out single clone 2A. The SIN1 knock out (KO) was performed by CRISPR/Cas9 using purified Cas9 protein, a human MAPKAP1 specific guide RNA, and nucleofection. The single clone was tested by western blotting using the SIN1 antibody (red), pAKT^{S473} antibody (green) around 60 kDa. GAPDH served as a loading control (green, 37kDa).
